# The mid-term and long-term effects of tourniquet use in total knee arthroplasty: systematic review

**DOI:** 10.1186/s40634-022-00471-1

**Published:** 2022-05-12

**Authors:** Wardah Rafaqat, Sudhesh Kumar, Tashfeen Ahmad, Zul Qarnain, Khalid Saeed Khan, Riaz Hussain Lakdawala

**Affiliations:** 1 Aga Khan University, Stadium Road, Karachi, Sindh 74800 Pakistan; 2 Department of Surgery, Aga Khan University, Stadium Road, Karachi, Sindh 74800 Pakistan; 3 Beatriz Galindo Programme, Department of Preventive Medicine and Public Health, University of Granada, Granada, Spain

**Keywords:** Tourniquet, Total knee arthroplasty, Total knee replacement, Long-term outcome, TKA

## Abstract

**Purpose:**

A tourniquet is routinely used during total knee arthroplasty (TKA) to reduce intra-operative hemorrhage, though surgery without a tourniquet is becoming popular. To address concerns about the effect of blood at cement interfaces on long-term implant stability, we conducted a systematic review among patients undergoing total knee arthroplasty to determine if TKA with a tourniquet, compared to TKA without a tourniquet or with reduced tourniquet duration, is associated with better mid-term and long-term implant stability.

**Methods:**

A literature search was conducted without language restriction in PubMed, Cochrane database and Web of Science from conception to 17th March, 2021. Prospective cohorts, randomized and observational, that compared tourniquet use with a control group, followed patients for 3 months or more and reported outcomes concerning implant stability, limb function, pain and inflammation. Article selection, quality assessment according to the Revised Cochrane risk assessment scale and Newcastle Ottawa Scale, and data extraction were conducted in duplicate. PROSPERO: CRD42020179020.

**Results:**

The search yielded 4868 articles, from which 16 randomized controlled trials (RCT) and four prospective cohort studies, evaluating outcomes of 1884 knees, were included. Eleven RCTs were evaluated to be low overall risk of bias, five RCTs had some concerns and four cohort studies were good quality. Few studies showed benefits of tourniquet use in mid-term implant stability (1/6), pain (1/11) and limb inflammation (1/5), and long-term implant stability (1/1). One study reported a significantly improved range of motion (1/14) while another reported significantly reduced quadriceps strength (1/6) in the tourniquet group. The remaining studies reported non-significant effect of tourniquet use.

**Conclusion:**

Although few studies indicated benefits of tourniquet use in mid-term pain, limb inflammation, implant loosening and function, and long-term implant loosening, the majority of studies report no significant advantage of tourniquet use in total knee arthroplasty.

**Supplementary Information:**

The online version contains supplementary material available at 10.1186/s40634-022-00471-1.

## Introduction

Total Knee Arthroplasty (TKA) is a major orthopedic intervention which is becoming more common as there is increase in the aging population and rates of obesity [5]. The main clinical indication for TKA is osteoarthritis, which accounts for 94–97% of operations [8]. A tourniquet is frequently though, not universally used in TKA to ensure bloodless visualization of structures and hence, reduce amount of blood loss intra and postoperatively [46]. It is thought to improve cementation by greater cement inter-digitation because of a dry cement bone interface [2, 13, 49]. However, the reduction in blood supply due to tourniquet application and the resultant ischemia is thought to increase risk of muscle damage, pain, swelling and cause slow recovery [1, 14, 21, 24, 26, 36, 37, 42, 43]. Thus, the long-term effectiveness of tourniquet use remains contested and guidelines regarding use of tourniquet in total knee arthroplasty have not yet been established.

To understand the effects better, there has been a recent increase in articles assessing mid-term and long-term outcomes of tourniquet use, especially implant stability. However, previous systematic reviews have not summarized such outcomes as they focus mostly on short-term effects and present conflicting evidence. Thus, some reviews report no significant difference in outcomes including pain, range of motion and blood loss in the tourniquet vs non-tourniquet group, others report a significant increase in pain, intraoperative blood loss and transfusion rates with tourniquet use [5, 23, 27, 29, 46, 47]. With respect to implant stability, one review reported an improvement due to tourniquet application but, remained limited to evidence from the immediate postoperative period, [29] while a recent Cochrane review excluded relevant evidence due to stringent restrictions on article type and outcome tool [3]. When evaluated for their reporting quality, [45] existing reviews also fail to mention registration of the review protocol prior to its commencement, [5, 22, 23, 29, 30, 46, 47, 53] conduct a completely comprehensive search strategy including grey literature or trial registries [5, 22, 46, 47, 53] or assess the impact of risk of bias when discussing the results of the review [5, 22, 46, 47]. Therefore, there is a need for a comprehensive, robust overview of the most recent literature.

We conducted a systematic review among patients undergoing total knee arthroplasty to determine if the procedure with a tourniquet, compared to the procedure without a tourniquet or with reduced tourniquet duration, is associated with better mid-term and long-term implant stability, pain, inflammation and function.

## Materials and methods

This systematic review was conducted after protocol registration (PROSPERO #CRD42020179020) and reported following the PRISMA statement [34].

### Search and selection

Randomized controlled Trials (RCT) and prospective cohort studies that compared patient outcomes after use of tourniquet in TKA with a control group and assessed outcomes after a follow-up of at least 3 months were included. Inclusion of RCTs and prospective cohort studies ensured that the review consisted of studies with a high level of evidence [25]. Acceptable control groups included a placebo as a sham tourniquet, no tourniquet, a different duration of tourniquet use or other measures to reduce blood loss, such as tranexamic acid. Studies with all types of thigh tourniquet (inflatable/ pneumatic or non-inflatable) that were used for any duration of the TKA were included. No restrictions regarding year of publication or language of the article were applied. Articles that did not mention receiving approval from an ethical review committee were excluded. Articles that only existed as registries and for which, the full article was not available were also excluded.

On 03/17/2021, the databases of PubMed, Web of Science, and Cochrane Library were systematically retrieved. The following keywords were used: TKR“ OR “total knee replacement” OR “total knee arthroplasty” OR “TKA“ AND “tourniquet” OR “pneumatic” OR “non inflatable” OR “non-inflatable” (Additional file 1: Appendix 1). The references of the included studies were also checked to find possible relevant articles. The search included registries of registered trials. The titles and abstracts of the citations were reviewed independently by two reviewers and full texts of the articles that either reviewer found relevant were acquired with the input of a library consultant, and assessed.

### Data extraction and study quality assessment

Data was extracted in duplicate. A third reviewer was consulted in cases of disagreements. Data on the following variables was extracted: study population (number, age, gender, BMI and disease of participants in each group), tourniquet use details (type, duration and pressure), study design, funding sources, conflict of interest and outcomes. Outcomes were further divided into early mid-term outcomes i.e., occurring after 3 months of surgery to less than 12 months, late mid-term outcomes i.e., 12 months to less than 60 months and long-term outcomes i.e., occurring after 60 months of surgery. When outcomes were recorded at multiple time points within a category, the greater follow-up time was selected. Primary outcomes consisted of implant stability, pain and lower limb function and while secondary outcomes consisted of any other outcomes that may have been recorded by the study. For studies that reported outcomes as graphs, web plot digitizer was used to read the graph accurately and extract data [41]. The graph was interpreted by two reviewers and an average of both readings was recorded.

The methodological quality of the included RCTs was evaluated independently by two reviewers based on the Cochrane Handbook for Systematic Reviews of Interventions, version 6.0 [18]. The following -item scales were assessed: random sequence generation (selection bias), allocation concealment (selection bias), blinding of the participants and personnel (performance bias), blinding of outcome assessments (detection bias) and selective reporting (reporting bias). Each of the items needed to be measured as “Yes” (low risk of bias), “No” (high risk of bias), or “Unclear” (unclear risk of bias). The risk of bias summary figure was obtained using the Robvis visualization tool [31]. The methodological quality of the cohort studies was assessed using the Newcastle Ottawa Scale [50]. The scale uses a star system to assess quality of studies based on selection, comparability of groups and ascertainment of outcome. Bias was assessed at the study level. Disagreements between the two reviewers in judgement of the quality of the study were settled by discussion and consultation with a third reviewer. The conflict of interest and source of funding reported by all the studies included was also recorded.

### Data synthesis

Sub-group analysis of studies with varying duration of tourniquet use was performed. Mean and standard deviation of the outcomes reported in the included studies was summarized in a table and the significance of the differences was recorded.

## Results

### Study selection

**Fig. 1:**
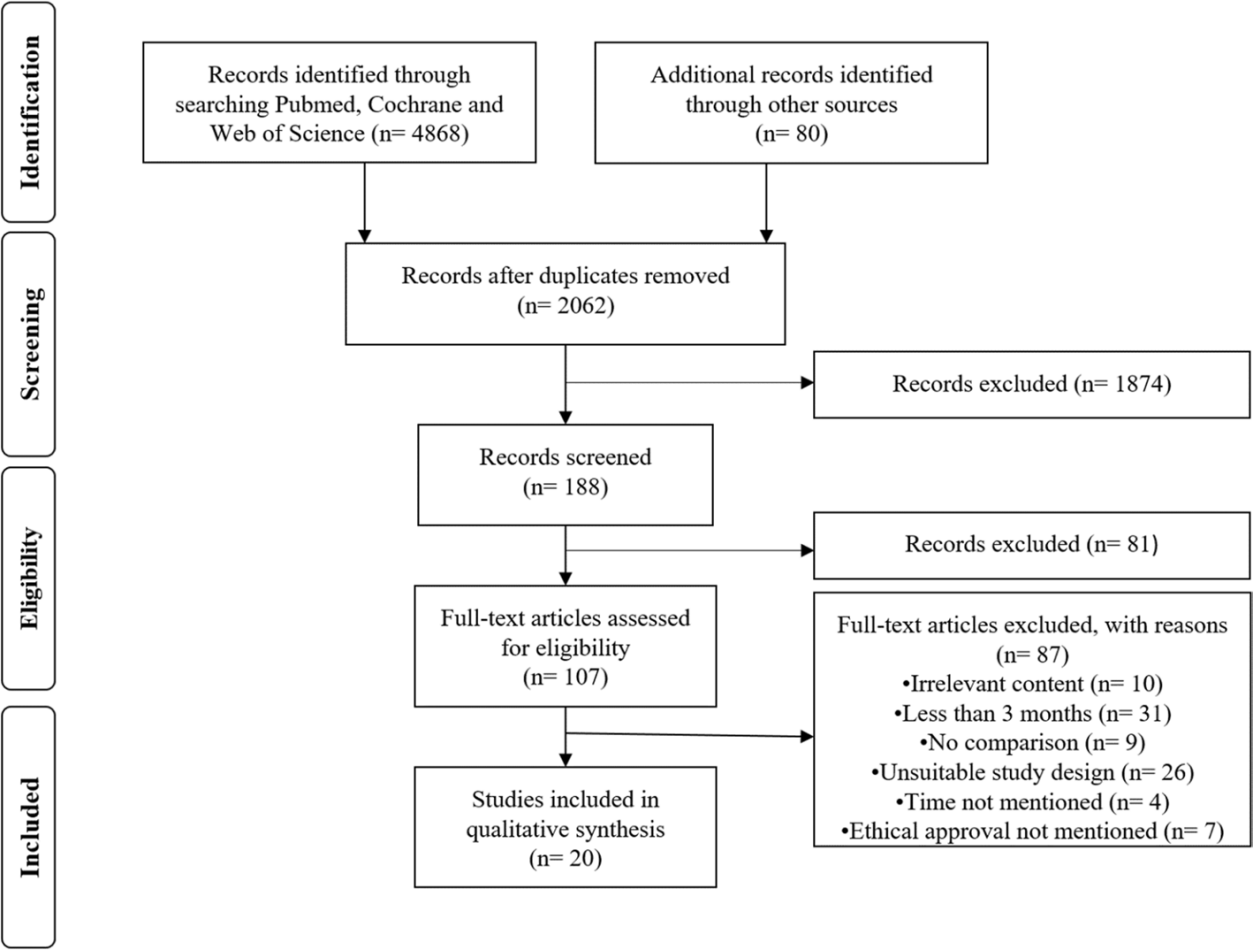
Flow chart for selection of studies for systematic review on long-term effects of tourniquet use in TKA

A total of 4868 articles were identified from literature databases and through reference and citation searches. After exclusion of duplicates, there were 2062 remaining, which were assessed for relevance by reviewing the title and abstract. One hundred eighty-eight articles were found relevant and the full text was available for 107 articles which were all in English despite absence of language restriction in the search and selection strategy. After the exclusion criteria were applied, a total of 20 articles were included in the final systematic review [4, 6, 7, 9–12, 15, 17, 19, 20, 28, 32, 35, 38, 40, 48, 51, 54, 55] (Fig. 1).

### Study characteristics

A total of 1884 subjects were undergoing total knee arthroplasty in the 20 included studies. The patients had a primary diagnosis of osteoarthritis and rheumatoid arthritis [4, 6, 7, 9–12, 15, 17, 19, 20, 28, 32, 35, 38, 40, 48, 51, 54, 55]. Tourniquet pressure was reported in all but one study and ranged between approximately 200–300 mmHg. The mean follow-up time was 12.2 months (Table 1) [4, 6, 7, 9–12, 15, 17, 19, 20, 28, 32, 35, 38, 40, 48, 51, 54, 55]. BMI, age and male:female ratio was reported by all studies. BMI ranged from 24.8–33 kg/m^2^ and mean age ranged from 62 to 73 years and male:female ratio ranged from 0.22 (13/59) to 9 (09/01) (Additional file 1: Appendix 2). There was no significant difference reported in baseline demographics between treatment groups.

**Table 1:** Characteristics of studies included in systematic review on mid-term and long-term effects of tourniquet use. (See additional characteristics in Additional file 1: Appendix 2)

Study Details	Study type^a^	Study Proc^b^	Disease^c^	Allocation of Intervention^d^			Tourniquet Details^e^			Sample Size^f^			Follow-up time (month)
				Group A	Group B	Group C	TQ press. (mm Hg) A/B/C	TQ time (min) A/B/C	TQ Type	Group A; loss to FU	Group B; loss to FU	Group C; loss to FU	
Ajnin, et al (2014) [4]	PCS	B/L TKA	OA/RA	TQ	NT		300	NI	NI	29; 0	29; 0		8
Ejaz, et al (2014) [12]	RCT	TKA	OA	TQ	NT		250	NI	NI	33; 0	31; 0		12
Hasanain, et al (2018) [15]	RCT	B/L TKA	OA/RA	LD	SD		100–150 abv. Sys. BP/	NI	Aut.	54; NI	54; NI		3
Huang, et al (2017) [19]	RCT	TKA	OA	TQ	TXA	TQ & TXA	100 abv. Sys. BP	NI	NI	50; 0	50; 0	50; 0	6
Jawhar, et al (2019) [20]	RCT	TKA	OA	TQ	NT		360	NI	Pneum	50; 8	49; 6		6
Liu, et al (2014) [28]	RCT	TKA	OA	TQ	NT		300	83/0	Pneum	10; 0	10; 0		12
Mittal, et al (2012) [32]	RCT	U/L TKA	OA	LD	SD		300	76.54 ± 15.1/ 22.5 ± 14.4	NI	34; 2	31; 5		12
Touzopoulos, et al (2019) [48]	PCS	TKA	OA	TQ	NT		350	NI	Pneum	50; 0	50; 0		36
Zhou et al (2017) [55]	RCT	U/L TKA	OA/RA	TQ	NT		NI	NI	NI	74; 6	74; 2		6
Alexandersson et al (2018) [6]	RCT	TKA	OA	TQ	NT		300	99 ± 15/ 0	Pneum	38; 1	43; 3		3
Molt, et al (2014) [35]	RCT	TKA	OA	TQ	NT		300	NI	NI	30; 8	30; 4		24
Dennis, et al (2015) [10]	RCT	B/L TKA	OA	TQ	NT		250	42/0	Pneum	14; 1	14; 1		3
Ejaz, et al (2015) [11]	RCT	TKA	OA	TQ	NT		250	NI	NI	33; 4	31; 3		24
Rathod, et al (2014) [40]	PCS	TKA	OA	LD	SD.		250–300	71.7 ± 8.9/ 36.8 ± 13.8	Padded	40; 3	40; 4		12
Ayik, et al (2020) [7]	RCT	U/L TKA	OA	TQ	NT		100 abv. Sys. BP	NI	Pneum	35; 3	35; 2		3
Chaudhry, et al (2020) [9]	RCT	TKA	OA	TQ	NT		250–300	NI	NI	148; 31	149; 26		6
Hedge, et al (2021) [17]	PCS	U/L TKA	OA	TQ	NT		250	30	Pneum	61; 0	61; 0		60
Pinsornsak, et al (2021) [38]	RCT	TKA	OA	TQ	TQ	TQ	75/100/150 abv. Sys. BP	65.1 ± 17.3/62.1 ± 18.8/ 59.3 ± 14.9	NI	50; 2	50; 2	50; 2	3
YiZ, et al (2021) [51]	RCT	TKA	OA	LD	SD	NT	100 abv. Sys. BP	61 ± 6.55/ 10.20 ± 1.92/0	NI	50; 0	50; 0	50; 0	3
Zhao, et al (2020) [54]	RCT	U/L TKA	OA	LD	SD	NT	100 abv. Sys. BP	72.70 ± 10.84/ 19.50 ± 4.43/ 0	NI	60; 0	60; 0	60; 0	3

^a^*PCS* Prospective Cohort Study, *RCT* Randomized Controlled Trial

^b^*Proc.* Procedure, *B/L* Bilateral, *TKA* Total Knee Arthroplasty, *U/L* Unilateral, *B/L* Bilateral

^c^*OA* Osteoarthritis, *RA* Rheumatoid Arthritis

^d^*TQ* Tourniquet, *NT* No Tourniquet, *LD* Long Duration, *SD* Short Duration, *TXA* Tranexamic Acid, *&* and

^e^*Abv* Above, *Sys* Systolic, *BP* Blood Pressure, *NI* No Information, *Press* Pressure, *min* minute, *Aut.* Automatic, *Pneum.* Pneumatic

^f^*FU* Follow-up

### Risk of bias within studies

**Fig. 2:**
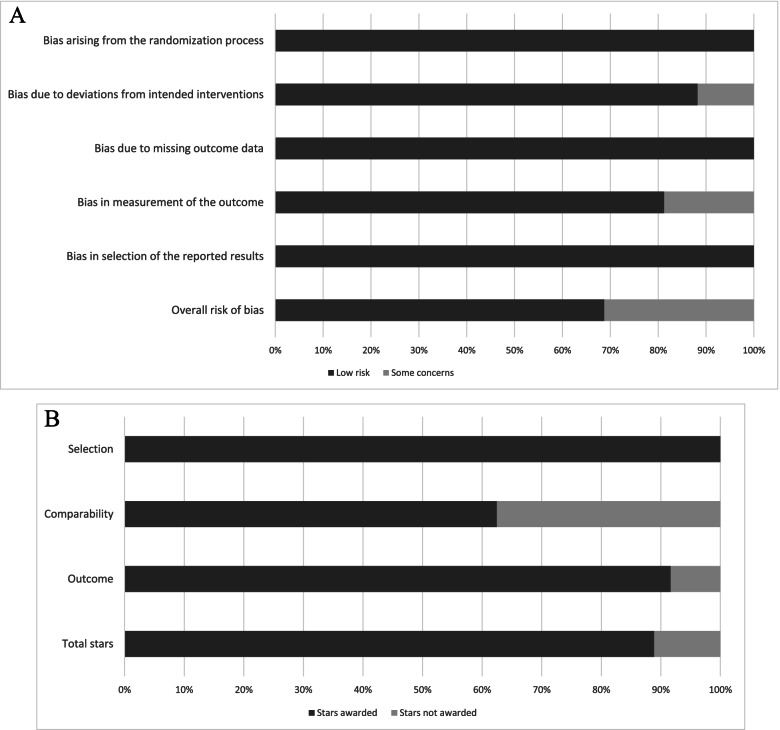
**A** Methodological quality assessed using Risk of Bias Assessment tool of RCTs included in review of long-term effects of tourniquet use in total knee arthroplasty (see additional details in Additional file 1: Appendix 3). **B** Methodological quality assessed using Newcastle Ottawa scale of prospective cohort studies included in review of long-term effects of tourniquet use in total knee arthroplasty (see additional details in Additional file 1: Appendix 4)

Eleven RCTs were determined as low overall risk of bias [6, 7, 9, 10, 19, 28, 32, 38, 51, 54, 55] and five RCTs were determined to have some overall concerns [11, 12, 15, 20, 33] according to the Revised Cochrane Risk Assessment Scale (Additional file 1: Appendix 3). There were some concerns present in mainly two domains: deviation from intended intervention and bias in measurement of the outcome in the RCTs with respectively, 11% and 19% of the values under these domains for all studies combined, belonging to “some concern” (Fig. 2A). All prospective cohort studies were reported as good quality according to the Newcastle Ottawa Scale Criteria [4, 17, 40, 48] (Fig. 2B). All studies received the maximum in the ‘selection’ criteria i.e., four stars. Three studies [4, 17, 40] were unable to control for, or adjust for disease severity of the participants and hence, only scored one star in ‘comparability’ and one study did not provide information regarding blinding of assessors and hence, only scored two stars in outcome assessment [4] (Additional file 1: Appendix 4).

Seven studies reported that no funding had not been received and declared no conflict of interest [6, 9, 16, 35, 38, 39, 54]. Seven studies did not mention whether funding had been received but, confirmed that no conflict of interest was present [4, 7, 11, 12, 20, 28, 48]. Three studies reported that funding had been received and reported conflict of interest [10, 17, 19] while, two studies reported that funding had been received and reported no conflict of interest [51, 56]. Finally, one study failed to report receiving funding or lack of conflict of interest [32].

### Implant stability

Implant stability was reported by seven studies [11, 12, 17, 20, 28, 35, 48] which included 529 knees and one study [17] reported long-term stability (Table 2). Implant stability was evaluated by measuring tibial cement penetration, progressive radiolucent lines (RLL) measured according to the Knee Society Roentgenographic Evaluation System (KSRES), percentage of radiolucency at the tibial bone–cement interface, translation and rotation of the tibia and recurrence rate. Notably, the studies did not include revision rate due to implant instability as a primary outcome or conduct statistical analysis of the outcome. Two studies found a significant increase in RLLs in the non-tourniquet groups. Touzopoulos, *et.al* found a significant decrease in percentage of radiolucency in anteroposterior (AP) view in the tourniquet group: (zone 1: 1.36 vs. 2.72 and zone 4: 1.14 vs. 2.67) and cumulative AP view (3.48 vs. 7.74) at 3 years [48]. Hedge, et al found a significant decrease in RLLs in the tourniquet group: AP view (zone 1: 0.33 vs. 1.53) and cumulative lateral view (0.79 vs. 5.58) at 5 years [17]. The remaining studies did not find a significant difference in outcomes in the treatment groups [11, 28, 35].

**Table 2:** Mid-term and late implant loosening in studies included in review on mid-term and long-term effects of tourniquet use

Study Details	Outcome meas. Tool	Early mid-term outcomes				Late mid-term outcomes				Long-term outcomes			
		Time point (month)	Outcome		***p*** value	Time point (month)	Score		***p*** value	Time point	Score		***p*** value
			Group A^**a**^	Group B^**b**^			Group A^**a**^	Group B^**b**^			Group A^**a**^	Group B^**b**^	
Ejaz, et al (2014) [12]	Revision rate					12	1/33	0/31	n.r.				
Liu, et al (2014) [28]	Tib. penet. (AP) (cm)					12							
	zone 1; zone 2; zone 3; zone 4; zone 5; zone 6; zone 7; zone 8; zone 9						0.17 ± 0.16; 0.29 ± 0.19; 0.32 ± 0.20; 0.21 ± 0.14; 0.28 ± 0.47; 0.97 ± 1.16; 0.30 ± 0.51; 0.29 ± 0.48; 0.29 ± 0.56	0.18 ± 0.09; 0.28 ± 0.36; 0.28 ± 0.27; 0.20 ± 0.30; 0.17 ± 0.31; 0.77 ± 1.34; 0.11 ± 0.18; 0.18 ± 0.41; 0.14 ± 0.36	n.s.				
	Tib. penet. (ML) (cm)					12							
	zone 1; zone 2; zone 3; zone 4; zone 5						0.21 ± 0.19; 0.20 ± 0.15; 0.73 ± 1.11; 0.24 ± 0.45; 0.33 ± 0.59	0.21 ± 0.19; 0.20 ± 0.18; 0.77 ± 1.40; 0.24 ± 0.46; 0.29 ± 0.62	n.s.				
	Fem. penet. (cm)					12							
	zone 1; zone 2; zone 3; zone 4; zone 5; zone 6; zone 7						0.12 ± 0.18; 0.22 ± 0.13; 0.14 ± 0.18; 0.05 ± 0.17; 0.22 ± 0.13; 0.10 ± 0.22; 0.15 ± 0.09	0.10 ± 0.2; 0.25 ± 0.24; 0.10 ± 0.14; 0.02 ± 0.06; 0.23 ± 0.15; 0.09 ± 0.23; 0.17 ± 0.07	n.s.				
Jawhar, et al (2019) [20]	Revision rate	6	3/50	2/49	n.r.								
Touzopoulos, et al (2019) [48]	RL KSRES					36							
	AP zone 1; zone 2; zone 3; zone 4; zone 5; zone 6; zone 7						1.36 ± 1.91; 1.01 ± 2.31; 1.20 ± 1.02; 1.14 ± 2.32; 0.06 ± 0.45; 0.0 ± 0.0; 0.0 ± 0.0	2.72 ± 2.88; 1.32 ± 2.09; 1.56 ± 2.83; 2.67 ± 3.27; 0.0 ± 0.0; 0.0 ± 0.0; 0.0 ± 0.0	0.007 n.s. n.s. 0.008 n.s. n.s. n.s.				
	Lat. zone 1; zone 2; zone 3						0.68 ± 1.7; 1.19 ± 2.58; 0.0 ± 0.0	1.43 ± 2.12; 1.87 ± 4.05; 0.05 ± 0.38	n.s. n.s. n.s.				
	Cum. AP						3.48 ± 4.69	7.74 ± 6.68	< 0.001				
	Cum. Lat.						1.76 ± 3.42	3.05 ± 4.23	n.s.				
	RL AP (%)						0.05 ± 0.07	0.11 ± 0.09	< 0.001				
	RL Lat.(%)						0.04 ± 0.07	0.07 ± 0.09	n.s.				
	Revision rate						0//50	0/50	n.s.				
Molt, et al (2014) [35]	Tib. Trans. (mm ± SD)	3				24							
	M-L		−0.10 ± 0.38	− 0.02 ± 0.21	n.s.		− 0.27 ± 0.71	− 0.13 ± 0.28	n.s.				
	C–Cl		0.00 ± 0.17	0.03 ± 0.28	n.s.		0.06 ± 0.34	0.05 ± 0.34	n.s.				
	PA		0.02 ± 0.15	0.04 ± 0.19	n.s.		−0.05 ± 0.50	0.05 ± 0.19	n.s.				
	Tib. Rot. (° ± SD)	3				24							
	Ant. Tilt		−0.02 ± 0.17	− 0.01 ± 0.16	n.s.		− 0.09 ± 0.28	−0.04 ± 0.23	n.s.				
	Int. Rot.		−0.01 ± 0.17	−0.04 ± 0.13	n.s.		−0.05 ± 0.35	0.01 ± 0.13	n.s.				
	Varus		0.01 ± 0.21	0.01 ± 0.20	n.s.		0.06 ± 0.48	−0.01 ± 0.29	n.s.				
	MTPM (mm ± SD)		0.37 ± 0.20	0.41 ± 0.18	n.s.		0.71 ± 0.64	0.53 ± 0.21	n.s.				
	Max MTPM (mm)	3	1.00	0.80	n.r.	24	2.84	1.19	n.r.				
Ejaz, et al (2015) [11]	Tib.trans (mm ± SD)	6				24							
	X trans.; Y trans.; Z trans.		0.02 ± 0.33; −0.04 ± 0.15; 0.05 ± 0.12	− 0.01 ± 0.22; − 0.02 ± 0.13; 0.03 ± 0.32	n.s.		0.03 ± 0.28; − 0.03 ± 0.18; − 0.02 ± 0.22	−0.04 ± .31; − 0.02 ± 0.27; 0.02 ± 0.28	n.s.				
	Tib. Rot. (° ± SD)	6				24							
	X rot.; Y rot.; Z rot.		0.02 ± 0.35; − 0.05 ± 0.95; 0.07 ± 0.30	−0.04 ± 0.50; 0.11 ± 0.83 0.03 ± 0.62	n.s.		−0.03 ± 0.47; − 0.12 ± 0.88; 0.04 ± 0.41	− 0.03 ± 0.31; − 0.08 ± 0.93; − 0.05 ± 0.58	n.s.				
	Mean MTPM (mm ± SD)	6	0.55 ± 0.42	0.51 ± 0.34	n.s.	24	0.47 ± 0.16	0.45 ± 0.21	n.s.	24			
	MTPM (med., min-max) (mm)	6	0.42, 0.23–1.01	0.42, 0.09–1	n.r.	24	0.47, 0.12–0.82	0.41, 0.09–0.98	n.r.	24			
	MTPM (LQ-UQ) (mm)	6	0.3–0.63	0.33–0.6	n.r.	24	0.38–0.56	0.31–0.58	n.r.	24			
Hedge, et al (2021) [17]	RL KSRES;									60			
	zone 1 (AP); zone 2 (AP); zone3M (AP); zone 3 L (AP); zone 5 (AP)										0.33 ± 1.56 0.19 ± 1.09 0 ± 0 0 ± 0 0 ± 0	1.53 ± 3.29 0.50 ± 1.70 1 ± 6 1 ± 6 0 ± 1	0.006 NI n.s. n.s. n.s.
	RL KSRES;									60			
	zone 1 (Lat); zone 2 (Lat); zone3A (Lat); zone 3P (Lat); Zone 5 (Lat); Cumulative										0.28 ± 1.08; 0 ± 0; 0 ± 0; 0 ± 0; 0 ± 0; 0.79 ± 2.28	0.85 ± 2.78; 0.50 ± 3.31; 0.53 ± 4.12; 0 ± 0; 0 ± 0; 5.58 ± 21.78	n.s.; n.s.; n.s.; n.s.; n.s.; 0.04
	Revision rate									60	0/61	2/61	n.r.

*Tib.* Tibial, *Penet.* Tibial Penetration, *AP* Anteroposterior, *ML* Mediolateral, *RL* Radiolucent Lines, *KSRES* Knee Society Roentgenographic Evaluation System, *Lat.* Lateral, Cum*.* Cumulative, *Rev. Surg.* Revision Surgery, *Tib. Rot.* Tibial Rotation, *Tib. Trans.* Tibial Translation, *C-CL* Cranio-Caudal, *PA* Posterior-Anterior, *Ant.* Anterior, *Int. rot.* Internal rotation, *MTPM* Maximum Total Point Motion, *LQ-UQ* Lower Quartile – Upper Quartile, *med* median, *min-max* minimum-maximum, *SD* Standard Deviation, *n.r.* not reported, *NI* no information

^a^participants with tourniquet use/ long duration tourniquet use

^b^participants without tourniquet use/ short duration use

### Functional outcomes

Functional outcomes appeared in 17 of the 20 included studies [4, 6, 7, 9, 10, 12, 15, 19, 20, 28, 32, 38, 40, 48, 51, 54, 55] including 1850 knees with no studies recording long-term outcomes (Table 3). Only two studies [10, 48] reported a statistically significant difference in functional outcomes. A study found a significant decrease in extension contracture in the tourniquet group at 36 months (1.51° vs. 5.61°) [48] while, another study found a significant decrease in quad strength in the tourniquet group at 3 months (127.6 Nm vs. 136.7 Nm) [10]. Functional outcomes were assessed by measuring range of motion, knee flexion, EMG activation signal, Quadriceps lag, stair ascent/descent time, straight leg raise, unilateral balance test, timed up and go test, muscle strength and force. A variety of tools including WOMAC, KOOS, NKSS, HSS and KSS were also used. Eleven studies used multiple tools to assess function [6, 7, 9, 10, 12, 20, 28, 32, 40, 48, 54, 55].

**Table 3:** Mid-term functional outcomes in studies included in review on mid-term and long-term effects of tourniquet use

Study Details	Outcome measurement tool	Early mid-term outcomes					Late mid-term outcomes			
		Time point (month)	Score			***p*** value	Time point (month)	Score		***p*** value
			Group A^**a**^	Group B^**b**^	Group C^**c**^			Group A^**a**^	Group B^**b**^	
Ajnin, et al (2014) [4]	Knee ROM (° ± SD)	8	106 ± 9	108 ± 10		n.s.				
	OKS ± SD	8	22 ± 3	21 ± 4		n.s.				
Ejaz, et al (2014) [12]	Knee ROM (° ± SD)	6	107 ± 11	108 ± 8.5		n.s.	12	113 ± 8	113 ± 8	n.s.
	KOOS-symptom; KOOS-ADL; KOOS-Sports/ Rec.	6	85.83; 85.83; 23.08	90.28; 86.64; 22.67		n.s.	12	91.90; 88.66; 23.08	92.71; 90.28; 23.48	n.s.
Hasanain, et al (2018) [15]	Knee Flex (°)	3	137.85	137.23		n.s.				
Huang, et al (2017) [19]	HSS	6	88.9	91.2	90.3	n.s.				
Jawhar, et al (2019) [20]	WOMAC-function; WOMAC- Stiff. ±SD	6	35 ± 19; 5 ± 3	45 ± 31; 5 ± 4		n.s.				
	OKS ± SD	6	25 ± 8	27 ± 9		n.s.				
	Conc. PF; Ecc. PF (N ± SD)	6	233 ± 7; 267 ± 11	220 ± 7; 253 ± 10		n.s.				
	Conc. WL; Ecc. WL (J ± SD)	6	65 ± 3; 71 ± 6	61 ± 3; 85 ± 14		n.s.				
	Conc. TWL; Ecc. TWL (J ± SD)	6	771 ± 52; 605 ± 41	723 ± 54; 628 ± 46		n.s.				
	Conc. MP; Ecc. MP (W ± SD)	6	42 ± 4; 24 ± 1	37 ± 3; 24 ± 1		n.s.				
Liu, et al (2014) [28]	EMG Act. signal^d^	6					12			
	VAM ± SD; VAL ± SD; RF ± SD		n.r.; 24,347 ± 7741; 20,998 ± 7741	27,618 ± 7741; 26,308 ± 7741; 29,414 ± 7741		n.s.		1631 ± 7028; 330 ± 3870; 0 ± 2441	495 ± 2903; 330 ± 2276; n.r.	n.s.
Mittal, et al (2012) [32]	Knee Flex. (°)	6	110.65	110.65		n.s.	12	114.41	115.91	n.s.
	Fixed Flex. (°)	6	5.47	8.37		n.s.	12	2.09	6.51	n.s.
	Quadriceps Lag (°)	6	4.92	3.22		n.s.	12	1.70	5.49	n.s.
	Stair ascent time (s)	6	7.13	7.13		n.s.	12	6.80	7.13	n.s.
	Stair descent time(s)	6	7.54	9.75		n.s.	12	6.87	9.09	n.s.
	OKS	6	39.21	37.37		n.s.	12	43.42	38.42	n.s.
Touzopoulos, et al (2019) [48]	NKSS-FA ± SD; NKSS-OM. ± SD						36	64.08 ± 16.06; 56.57 ± 4.92	66.04 ± 15.62; 56.81 ± 7.02	n.s. n.s.
	Total ROM (° ± SD)						36	99.51 ± 8.14	99.69 ± 8.65	n.s.
	Max. flex. (° ± SD)						36	101.86 ± 7.94	101.35 ± 8.55	n.s.
	Ext. Cont. (° ± SD)						36	1.51 ± 4.54	5.61 ± 5.86	< 0.001
Zhou, et al (2017) [55]	HSS ± SD	3	82.5 ± 4.5	81.6 ± 4.4		n.s.				
		6	89.8 ± 4.9	90.7 ± 4.5		n.s.				
	Knee ROM (°)	3	128.19	127.51						
		6	128.52	128.19						
Alexandersson, et al (2018) [6]	Knee flex; knee ext. (°)	3	107.1; 5.9	109.4; 5.6		n.s.				
	Straight Leg Raise(°)	3	37	35		n.s.				
	TUG Test (s)	3	11.2	10.1		n.s.				
Dennis, et al (2015) [10]	Knee flex.; knee ext. (° ± SD)	3	120.88 ± 8.19; 3.00 ± 2.71	122.69 ± 7.25; 2.46 ± 1.58		n.s.				
	HS St. (Nm ± SD)	3	84.84 ± 34.75	84.34 ± 36.01		n.s.				
	Quad Act. (% ± SD)	3	88.35 ± 10.55	87.02 ± 8.66		n.s.				
	Quad St. (Nm ± SD)	3	127.66 ± 43.75	136.74 ± 47.75		0.03				
	UBT (s ± SD)	3	47.77 ± 18.78	52.26 ± 15.68		n.s.				
Rathod, et al (2014) [40]	Knee flex. (° ± SD)	3	106 ± 15	109 ± 12		n.s.	12	114 ± 8	115 ± 8	n.s.
	KSSC ± SD	3	73.5 ± 11.9	71.4 ± 15.2		n.s.	12	87.9 ± 7.5	86.8 ± 9.1	n.s.
	Quad st. (Nm ± SD)	3	97 ± 42	105 ± 38		n.s.	12	116 ± 43	119 ± 42	n.s.
	KSSF ± SD	3	81.1 ± 9.8	79.8 ± 11.7		n.s.	12	85.6 ± 12.4	84.7 ± 11.3	n.s.
Ayik, et al (2020) [7]	Quad torque (Nm ± SD)	3	74 ± 29	68 ± 24		n.s.				
	Hamstring torque (Nm ± SD)	3	65 ± 18	57 ± 8		n.s.				
	Quad work (Nm ± SD)	3	85 ± 25	77 ± 31		n.s.				
	Hamstring work (Nm ± SD)	3	61 ± 18	54 ± 16		n.s.				
	KSS-knee score ± SD	3	79 ± 13	76 ± 12		n.s.				
	KSS-func. Score ± SD	3	79 ± 19	76 ± 20		n.s.				
	Total ROM ± SD	3	118 ± 10	115 ± 13		n.s.				
Chaudhry, et al (2020) [9]	OKS ± SD	6	39.02 ± 1.21	39.14 ± 1.20		n.s.				
	Knee ROM (° ± SD)	6	105.88 ± 3.608	106.74 ± 3.203		n.s.				
Pinsornsak, et al (2021) [38]	Knee flex. (° ± SD)	3	114.2 ± 12.1	114.7 ± 10.4	114.8 ± 8.4	n.s.				
YiZ, et al (2021) [51]	Knee ROM. (° ± SD)	3	111 ± 9	111 ± 9	112 ± 8	n.s.				
Zhao, et al (2020) [54]	AKSS ± SD	3	88.60 ± 2.35	89.15 ± 1.65	90.30 ± 1.74	n.s.				
	Quad st. (grade ± SD	3	4.07 ± 0.29	4.22 ± 0.26	4.43 ± 0.27	n.s.				
	Knee ROM (° ± SD)	3	122.00 ± 10.09	124.75 ± 9.93	126.91 ± 8.20	n.s.				

*Ecc.* Eccentric, *Conc.* Concentric, *TWL* Total Work Load, *WL* Work Load, *ROM* Range of Motion, *OKS* Oxford Knee Score, *KOOS* Knee Injury and Osteoarthritis Outcome Score, *ADL* Activities of Daily Living, *HSS* Hospital for Special Surgery knee score, *WOMAC* Western Ontario and McMaster Universities Osteoarthritis Index, *Func.* Function, *SD* Standard Deviation, *Conc.* Concentric, *PF* Peak Force, *WL* Work Load, *MP* Muscle Power, *EMG* Electromyography, *VAM* Vastus Medialis, *VAL* Vastus Lateralis, *RF* Rectus Femoris, *NKSS* New Knee Society Score, *FA* Functional Activities, *OM* Objective Measures, *Max.* Maximum, *Ext.* Extension, *Cont.* Contracture, *TUG test* Timed Up and Go test, *HS St.* Hamstring Strength, *UBT* Unilateral Balance Test, *KSSC* Knee Society Score Clinical, *Quad St.* Quadriceps Strength, *KSSF* Knee Society Score Functional, *n.s.* non-significant, *Flex* Flexion, *Stiff.* Stiffness, *Act.* Activation, *n.r.* not reported, *AKSS* American Knee Society Score, *EQ-5D* EuroQol 5-Dimensions

^a^tourniquet use/ long duration tourniquet use

^b^tourniquet use/ short duration use

^c^tourniquet use with tranexamic acid

^d^unit not reported

### Pain

Pain was reported in 11 studies [6, 7, 9, 10, 12, 15, 19, 20, 40, 48, 55] with 1227 knees (Table 4). Of the studies reporting pain, only one study reported a statistically significant increase in pain in the non-tourniquet group at 3 months, measured using the visual analogue scale (2.9 vs. 4.7) [6]. A variety of tools were used for the assessment of pain including the Western Ontario and McMaster Universities Osteoarthritis Index Pain score, Knee Injury and Osteoarthritis Outcome Score, Visual Analog Scale (VAS), Oxford Knee Score, Numeric Analog Scale, New Knee Society Score (NKSS), Hospital for Special Surgery knee score; Numeric Pain Rating Scale, Knee Society Score, Clinical, Short Form 36 and Physical Component Score with the most common being the VAS [6, 10, 12, 15, 19, 20, 40, 48, 55]. The VAS was used by four studies [15, 19, 40, 55] and scores ranged from to 0.14–4.7. Three studies used multiple tools to assess pain [20, 40, 55].

**Table 4:** Mid-term pain outcomes in studies included in review on mid-term and long-term effects of tourniquet use

Study Details	Outcome measurement tool	Mid-term outcomes								
		Time point (month)	Score			***p*** value	Time point (month)	Score		***p*** value
			Group A^**a**^	Group B^**b**^	Group C^c^			Group A^**a**^	Group B^**b**^	
Ejaz, et al (2014) [12]	KOOS-Pain	6	88.66	89.07		n.s.	12	91.09	92.31	n.s.
Hasanain, et al (2018) [15]	VAS	3	1.96	1.77		n.s.				
Huang, et al (2017) [19]	VAS ± SD	3	0.64 ± 0.63	0.48 ± 0.50	0.52 ± 0.54	n.s.				
		6	0.40 ± 0.73	0.32 ± 0.47	0.36 ± 0.48	n.s.				
Jawhar, et al (2019) [20]	WOMAC Pain ± SD	6	10 ± 7.04	13 ± 8.97		n.s.				
	OKS ± SD	6	25 ± 7.99	27 ± 8.92		n.s.				
	NAS-Pain ± SD	6	0.9 ± 0.2	1.2 ± 0.3		n.s.				
Touzopoulos, et al (2019) [48]	NKSS-symptoms ± SD						36	19.43 ± 1.74	19 ± 2.12	n.s.
Zhou, et al (2017) [55]	HSS ± SD	3	82.5 ± 4.5	81.6 ± 4.4		n.s.				
		6	89.8 ± 4.9	90.7 ± 4.5		n.s.				
	VAS (Thigh Pain) ± SD	3	0.16 ± 0.65	0.16 ± 0.65						
		6	0.14 ± 0.62	0.14 ± 0.62						
Alexandersson, et al (2018) [6]	VAS	3	2.9	4.7		< 0.05				
Dennis, et al (2015) [10]	NRPS ± SD	3	2.56 ± 2.14	2.32 ± 2.07		n.c.				
Rathod, et al (2014) [40]	VAS ± SD	3	2.7 ± 1.9	2.8 ± 1.8		n.s.	12	0.8 ± 1.4	1.1 ± 1.2	n.s.
	KSSC ± SD	3	73.5 ± 11.9	71.4 ± 15.2		n.s.	12	87.9 ± 7.5	86.8 ± 9.1	n.s.
	SF-36 PCS ± SD	3	40.72 ± 7.75	42.79 ± 8.31		n.s.	12	49.58 ± 7.34	49.55 ± 8.28	n.s.
Ayik, et al (2020) [7]	VAS ± SD	3	3 ± 1.3	3 ± 1.28		n.s.				
Chaudhry, et al (2020) [9]	NPRS ± SD	6	2.66 ± 018	2.60 ± 0.21		n.s.				
	VAS satisfaction ± SD	6	73.05 ± 11.84	70.23 ± 16.85		n.s.				

*KOOS* Knee Injury and Osteoarthritis Outcome Score, *VAS* Visual Analog Scale, *SD* Standard Deviation, *WOMAC* Western Ontario and McMaster Universities Osteoarthritis Index, *OKS* Oxford Knee Score, *NAS* Numeric Analog Scale, *NKSS* New Knee Society Score, *HSS* Hospital for Special Surgery knee score, *NRPS* Numeric Pain Rating Scale, *KSSC* Knee Society Score Clinical, *SF-36* Short Form 36, *PCS* Physical Component Score, *n.s.* non-significant, *n.c.* not calculated

^a^tourniquet use/ long duration tourniquet use

^b^tourniquet use/ short duration use

^c^tourniquet use with tranexamic acid

### Other outcomes

Other outcomes were reported by 10 studies including 973 knees [6, 9, 10, 12, 19, 20, 28, 35, 40, 48] (Table 5). The outcomes and outcome measurement tools included limb inflammation (limb circumference), limb alignment (Hip-knee-ankle index), patient satisfaction (patient satisfaction scale, Knee Injury and Osteoarthritis Outcome Score (KOOS), EuroQol, Manusco, Knee Society Score), patient anxiety (Hospital Anxiety and Depression Score, quality of life (KOOS and 12-Item Short Form Health Survey), and general health (Physical Component Score, Mental Component Score) with limb inflammation [6, 10, 28, 40] and patient satisfaction [19, 20, 48] being the most frequently reported outcomes. A study reported a significant increase in knee circumference in the non-tourniquet group at 12 months (41.27 vs. 42.62) [28]. There were no other reported significant differences in other outcomes.

**Table 5:** Mid-term other outcomes in studies included in review on mid-term and long-term effects of tourniquet use

Study Details	Outcome measured	Outcome measurement tool	Mid-term outcomes								
			Time point (month)	Score			***p*** value	Time point (month)	Score		***p*** value
				Group A^**a**^	Group B^**b**^	Group C^**c**^			Group A^**a**^	Group B^**b**^	
Ejaz, et al (2014) [12]	QOL	KOOS-QOL	6	81.377	80.972		n.s.	12	83.81	84.21	n.s.
Huang, et al (2017) [19]	Pt. Sat.	Sat.	3								
		Ext. Sat.		19	34	32	n.s.				
		V. Sat.		16	12	15					
		SW Sat.		9	4	2					
		N. sat. nor dissat.		5	0	1					
		SW Dissat.		1	0	0					
		V. Dissat.		0	0	0					
		Ext. Sat.	6	22	34	33	n.s.				
		V. Sat.		20	13	14					
		SW Sat.		4	3	2					
		N. sat. nor dissat.		3	0	1					
		SW Dissat.		1	0	0					
		V. Dissat.		0	0	0					
Jawhar, et al (2019) [20]	Pt. Dep.	HADS Depression	6	3 ± 3.07	3 ± 4		n.s.				
	Pt. Sat.	Mancuso Score	6	34 ± 8.36	36 ± 16.11		n.s.				
	Pt. SF hth.	EQ VAS	6	74 ± 17.40	75 ± 17.47		n.s.				
	HR QOL	EQ 5D Index	6	0.91 ± 0.08	0.91 ± 0.08		n.s.				
	Pt. Anxiety	HADS-Anxiety	6	3 ± 2.91	4 ± 3.93		n.s.				
Liu, et al (2014) [28]	Limb Inf.	Knee Circum. (cm)	6	44.16 ± 0.66	40.47 ± 0.60		n.s.	12	41.27 ± 2.31	42.62 ± 5.79	sig.
	Limb Inf.	Thigh Circum. (cm)	6	47.90 ± 0.79	41.23 ± 1.03		n.s.	12	48.22 ± 10.96	46.79 ± 10.72	n.s.
Touzopoulos, et al (2019) [48]	Pt. Sat.	KSS-Sat.						36	35.57 ± 5.48	34.65 ± 5.82	n.s.
	Pt. Expec.	KSS- Expec.						36	9.04 ± 1.64	9.08 ± 1.46	n.s.
Alexandersson, et al (2018) [6]	Limb Inf.	Knee Circum. (cm)	3	44.6	44.7		n.s.				
Molt, et al (2014) [35]	Limb Al.	HKA index (°)	3	179 ± 4	179 ± 3		n.s.				
Dennis, et al (2015) [10]	Limb Inf.	Thigh Girth (cm)	3	46.05 ± 5.57	45.97 ± 6.14		n.s.				
	Limb Inf.	Calf Girth (cm)	3	36.31 ± 3.68	36.16 ± 3.87		n.s.				
	Limb Inf.	Knee Girth (cm)	3	43.9 ± 4.42	43.53 ± 4.29		n.s.				
Rathod, et al (2014) [40]	Limb Inf	KSSF	3	42.6 ± 5.1	43.1 ± 3.9		n.s.	12	41.7 ± 3.9	42.9 ± 6.9	n.s.
	Gen. hth.	PCS	3	40.72 ± 7.75	42.79 ± 8.31		n.s.	12	49.58 ± 7.34	49.55 ± 8.28	n.s.
	Gen. hth.	MCS	3	47.96 ± 11.53	48.8 ± 11.11		n.s.	12	54.45 ± 7.51	53.68 ± 9.36	n.s.
	Limb Inf.	MPC (cm)	3	106 ± 15	109 ± 12		n.s.	12	116 ± 43	119 ± 42	n.s.
Chaudhry, et al (2020) [9]	Pt. QOL	SF-12 ± SD	6	39.32 ± 1.471	39.35 ± 2.102		n.s.				

*QOL* Quality of Life, *Pt.* Patient, *Sat.* Satisfaction, *Ext.* Extreme, *V* Very, *SW* Somewhat, *N. sat. not dissat.* Neither satisfied nor dissatisfied, *Dep.* Depression, *SF hth.* Self Rated Health, *HR* Health Related, *Circum* Circumference, *HADS* Hospital Anxiety and Depression Score, *EQ* EuroQol, *5D* 5 Dimensions, *Gen.* General, *KSSF* Knee Society Score Functional, *PCS* Physical Component Score, *MCS* Mental Component Score, *MPC* Mid-patellar Circumference, *HKA Index* Hip Knee Ankle Index, *SF-12* 12-Item Short Form Health Survey, *n.s.* non-significant, *Inf.* Inflammation, *Al.* Alignment, *sig.* significant

^a^tourniquet use/ long duration tourniquet use

^b^tourniquet use/ short duration use

^c^tourniquet use with tranexamic acid

## Subset analysis on difference in tourniquet duration

A subset of five studies compared long duration of tourniquet application with a shorter duration of application [15, 32, 40, 51, 54]. The long duration group consisted of application of tourniquet throughout the procedure (incision to closure of wound) while, the short duration group consisted of application of tourniquet during cementation only (inflated during component cementing and deflated after cement hardened). No significant differences were observed in pain [15, 40], function [15, 32, 40, 51, 54] and limb inflammation [40] between the long duration and short duration group.

## Discussion

In this review, we found that there was no significant difference in implant stability and mid-term pain and function reported in the majority of studies. The studies that reported a significant difference, showed benefits of tourniquet use in long-term implant stability and mid-term pain management and limb inflammation. However, no homogenous conclusion was reached with regards to functional outcomes. A study reported a better functional outcome in the tourniquet group while, another reported a better outcome in the non-tourniquet group.

One of the strengths of our review is that we looked at mid-term and long-term implant stability, as these outcomes have been insufficiently analyzed in previous reviews, a lacking that has been identified by a recent Cochrane review conducted by Ahmed, et al. [3]. However, the recent increase in comparative studies on the topic allowed us to comprehensively summarize evidence on it. Implant stability is a significant outcome since it can be used to assess risk for revision surgery. Our review uses a variety of outcome measures including tibial cement penetration, progressive radiolucent lines (RLL), percentage of radiolucency at the tibial bone–cement interface, translation and rotation of the tibia and revision rate to capture all the available evidence present on implant loosening.

Another strength of our review is that we focused on outcomes that were measured after an extended period of follow-up to offer a complete perspective on the effects of tourniquet use, an aspect that previous reviews have not focused on. The extended follow-up period is important when faced with conflicting evidence regarding short-term outcomes of tourniquet use as it doesn’t only offer another perspective, but also helps understand the duration for which differences that may be present in short-term outcomes persist. It is also pertinent when assessing certain outcomes such as, implant stability. RLLs, a standardized method for measuring implant stability, can only be assessed properly at a long-term follow-up visit. They may be present in the immediate postoperative X-ray due to poor cementation technique but, while such lines can facilitate the entry of debris to the cement–bone interface, they may be non-progressive and hence, not affect the fixation of the implant [44, 48]. The included studies assessing implant loosening especially, had follow-up periods extending up to 5 years. Therefore, the information summarized in our review will be useful for guidelines regarding the use of tourniquet in TKA in the future.

We also identified that the included studies did not include revision surgery, a clinically significant outcome, as a primary measurement tool of long-term implant stability and did not conduct statistical analysis even when revision rate was reported. This is likely due to the follow-up duration not being sufficient to capture rate of revision surgery. The cases of early revision surgery that were reported were primarily due to operative factors such as, lack of intraoperative recognition of impaired subchondral tibial bone quality due to cyst [12], or immediate postoperative factors including, surgical site infections and hematoma formation [20]. Among the studies included, only Hedge, et al. was able to partially capture revision surgery due to aseptic implant loosening through a follow-up of 5 years [17]. It is necessary that further studies focus on revision surgery as a measure of implant failure, as it is a critical outcome, and follow-up patients for periods exceeding 10 years to capture it adequately.

The systematic review consisted of low risk RCTs and good quality prospective cohort studies. Therefore, a significant effect of bias was not expected on the results. Restricting the study design of included studies to RCTs and prospective cohorts allowed inclusion of greater level of evidence [25] but, limited the sample size of available studies. We sought to remedy this by conducting an extensive search strategy to identify and review all potentially relevant papers, and by including all the outcomes recorded at, or after 3 months and reporting them as other outcomes. Including these outcomes, provided a holistic view into the impact of tourniquet use on the patient’s quality of life, satisfaction, and mental and physical general health, as well as additional limb parameters including inflammation and alignment. Very rarely have previous reviews summarized evidence on these outcomes and never with a focus on long-term follow-up. The long-term follow-up is essential when assessing outcomes like patient quality of life (QOL) as they provide a true picture of how the QOL is impacted once the recovery from the procedure is complete. There was prominent heterogeneity in the tools used to measure functional outcomes and pain which can be explained by the presence of various validated tools to assess these outcomes. In contrast, tools used to assess implant loosening were relatively homogenous.

A small subset of our population compared the impact of the duration of tourniquet use. Including these studies allowed us to assess whether the technique of tourniquet application needs to be altered. There were no significant differences found in the long-term outcomes of these studies [15, 32, 40, 51, 54]. Our findings are pertinent in light of this conflicting evidence present currently. A previous review evaluating the impact of timing of tourniquet release found no significant difference in blood loss, hemoglobin levels and blood transfusions [53] while, another review showed increased perioperative blood loss and a decreased risk of complications with use of tourniquet for a shorter duration [52].

The included studies contained a detailed description of the steps taken to standardize the TKA and to allow comparability of outcomes between the intervention and control group. However, details of tourniquet use were generally sparse. While, all studies except one reported the tourniquet pressure and there was homogeneity in the pressure used between studies, only a few studies [6, 7, 10, 15, 17, 20, 28, 40, 48] reported the type of tourniquet used and even fewer reported the duration of tourniquet application. Therefore, a limitation of this review was that it was not possible to fully understand the effect of differences in the tourniquet application technique between studies on outcomes.

The size of the groups included in each category (early mid-term, late mid-term and long-term) was not well balanced, in particular only 4/20 studies include follow-up more than 12 months. Moreover, only 7/20 studies assessed implant stability. Therefore, while this review aimed to capture long-term outcomes, it was limited in its ability to do so especially, implant stability. The findings of this review highlight the necessity for studies to assess implant stability with follow-up period extending to at least 5 years.

While short-term impacts of tourniquet use in TKA have been studied frequently, there is lack of analysis of the mid-term and long-term outcomes of tourniquet use in TKA. Evidence for short-term outcomes may rely more heavily on the technique of tourniquet use and expertise of the surgeon instead of tourniquet use. Therefore, it remains largely inconclusive. In the past two decades, comparative studies have increasingly focused on long-term outcomes of tourniquet use in TKA, especially implant stability, and this review provides an updated summary of the results of these studies. Hence, it provides an additional perspective to judge the effects of tourniquet use.

## Conclusion

Although few studies indicated benefits of tourniquet use in mid-term pain, limb inflammation, implant loosening and function, and long-term implant loosening, the majority of studies report no significant advantage of tourniquet use in total knee arthroplasty. Our review also highlights a gap in literature regarding studies assessing impact of tourniquet use on rates of revision surgery due to implant instability in the long term. To examine this important and clinically significant outcome, further studies with a longer duration of follow-up, exceeding 10 years, may be required.

## Supplementary Information

**Additional file 1: Appendix 1.** Search strategy for studies on long-term effects of tourniquet use in total knee arthroplasty. **Appendix 2.** Population demographics of studies included in the systematic review on long-term complications of tourniquet use. **Appendix 3.** Quality assessment of RCTs using Risk of Bias Assessment tool for review assessing the long-term effects of tourniquet use in TKA. **Appendix 4.** Quality assessment of cohort studies using Newcastle-Ottawa scale for review assessing the long-term effects of tourniquet use in TKA.

## Data Availability

All of the collected data has been included in the tables present in manuscript.
