# Supplementary material for: The mid-term and long-term effects of tourniquet use in total knee arthroplasty: systematic review
Source: J Exp Orthop. 2022 May 12;9:42. doi: 10.1186/s40634-022-00471-1 (PMC9098769; doi:10.1186/s40634-022-00471-1)
Supplement: Supplementary file 1 — Additional file 1: Appendix 1. Search strategy for studies on long-term effects of tourniquet use in total knee arthroplasty. Appendix 2. Population demographics of studies included in the systematic review on long-term complications of tourniquet use. Appendix 3. Quality assessment of RCTs using Risk of Bias Assessment tool for review assessing the long-term effects of tourniquet use in TKA. Appendix 4. Quality assessment of cohort studies using Newcastle-Ottawa scale for review assessing the long-term effects of tourniquet use in TKA. [file 40634_2022_471_MOESM1_ESM.docx]

Appendix 1: Search strategy for studies on long-term effects of tourniquet use in total knee arthroplasty

((tourniquet [MeSH]) OR (tourniquet) OR (pneumatic) OR (non-inflatable) OR (non inflatable)) AND ((total knee arthroplasty [MeSH]) OR (total knee arthroplasty) OR (TKR) OR (TKA) OR (total knee replacement [MeSH]) OR (total knee replacement))

The following are the search results from databases used:

Pubmed

| 1 | ((tourniquet [MeSH]) OR (tourniquet)) | 7,700 |
| --- | --- | --- |
| 2 | (pneumatic) | 11,077 |
| 3 | ((non-inflatable) OR (non inflatable)) | 3,078 |
| 4 | ((total knee arthroplasty [MeSH]) OR (total knee arthroplasty)) | 36,012 |
| 5 | (total knee replacement [MeSH]) OR (total knee replacement)) | 31,988 |
| 6 | (TKA) | 12,445 |
| 7 | (TKR) | 2,259 |
| 8 | ((tourniquet [MeSH]) OR (tourniquet) OR (pneumatic) OR (non-inflatable) OR (non inflatable)) AND ((total knee arthroplasty [MeSH]) OR (total knee arthroplasty) OR (TKR) OR (TKA) OR (total knee replacement [MeSH]) OR (total knee replacement)) | 1,052 |

Web of Science

| 1 | (tourniquet) | 6,067 |
| --- | --- | --- |
| 2 | (pneumatic) | 29,649 |
| 3 | ((non-inflatable) OR (non inflatable)) | 359 |
| 4 | (total knee arthroplasty) | 33,646 |
| 5 | (total knee replacement) | 22,833 |
| 6 | (TKA) | 11,212 |
| 7 | (TKR) | 2,246 |
| 8 | (total knee arthroplasty) AND ((tourniquet) OR (pneumatic) OR (non-inflatable) OR (non inflatable)) | 1,051 |
| 9 | (total knee replacement) AND ((tourniquet) OR (pneumatic) OR (non-inflatable) OR (non inflatable)) | 711 |
| 10 | (TKA) AND ((tourniquet) OR (pneumatic) OR (non-inflatable) OR (non inflatable)) | 517 |
| 11 | (TKR) AND ((tourniquet) OR (pneumatic) OR (non-inflatable) OR (non inflatable)) | 69 |
| 12 | ((total knee arthroplasty) OR (TKR) OR (TKA) OR (total knee replacement)) AND ((tourniquet) OR (pneumatic) OR (non-inflatable) OR (non inflatable)) | 1,157 |

[Cochrane Central Register of Controlled Trials](https://www.cochranelibrary.com/)

| 1 | (total knee arthroplasty) | 6,663 |
| --- | --- | --- |
| 2 | (total knee replacement) | 4,871 |
| 3 | (TKR) | 667 |
| 4 | (TKA) | 2,933 |
| 5 | (tourniquet) | 2,003 |
| 6 | (pneumatic) | 2,023 |
| 7 | (non-inflatable) | 31 |
|  | (non inflatable)) | 166 |
| 8 | ((total knee arthroplasty) OR (TKR) OR (TKA) OR (total knee replacement)) | 7,908 |
| 9 | ((tourniquet) OR (pneumatic) OR (non-inflatable) OR (non inflatable)) | 3,945 |
| 10 | ((total knee arthroplasty) OR (TKR) OR (TKA) OR (total knee replacement)) AND ((tourniquet) OR (pneumatic) OR (non-inflatable) OR (non inflatable)) | 622 |

Appendix 2: Population demographics of studies included in the systematic review on long-term complications of tourniquet use.

| Study Details | Mean age (year ± SD) | | | m/f ratio | | | BMI (kg/m^2^ ± SD) | | |
| --- | --- | --- | --- | --- | --- | --- | --- | --- | --- |
|  | **Group A^1^** | **Group B^2^** | **Group C^3^** | **Group A^1^** | **Group B^2^** | **Group C^3^** | **Group A^1^** | **Group B^2^** | **Group C^3^** |
| Ajnin, *et al* (2014)[4] | 73 | 73 |  | NI | NI |  | 33.0 | 33.0 |  |
| Ejaz, *et al* (2014)[15] | 68.0 ± 8.4 | 68.0 ± 7.8 |  | 18/15 | 17/14 |  | 25 ± 2.0 | 25 ± 2.5 |  |
| Hasanain, *et al* (2018)[18]* | 62.9 ± 7.5 | 62.9 ± 7.5 |  | 20/34 | 20/34 |  | 32.4 ± 4.9 | 32.4 ± 4.9 |  |
| Huang, *et al* (2017)[23] | 65.8 ± 6.3 | 65.1 ± 6.8 | 66.2 ± 8.3 | 15/35 | 16/34 | 18/32 | 24.7 ± 1.3 | 24.4 ± 1.5 | 25.1 ± 1.5 |
| Jawhar, *et al* (2019)[24] | 69.3 ± 7.4 | 68.3 ± 7.8 |  | 17/33 | 19/30 |  | 31.9 ± 6.0 | 31.4 ± 5.5 |  |
| Liu, *et al* (2014)[32] | 67.0 | 70.0 |  | 07/03 | 09/01 |  | 25.6 | 27.1 |  |
| Mittal, *et al* (2012)[36] | 66.8 ± 8.4 | 67.5 ± 8.9 |  | 09/25 | 06/25 |  | 32.6 ± 5.6 | 32.5 ± 5.6 |  |
| Touzopoulos, *et al* (2019)[52] | 70.7 ± 6.6 | 69.9 ± 6.9 |  | 42/8 | 42/8 |  | 31.0 ± 5.4 | 31.3 ± 4.0 |  |
| Zhou *et al* (2017)[59] | 69.1 ± 7.6 | 66.8 ± 8.6 |  | 07/61 | 13/59 |  | 25.7 ± 3.4 | 26.1 ± 4.1 |  |
| Alexandersson *et al* (2018)[7] | 68.0 ± 7.4 | 69.7 ± 6.4 |  | 18/20 | 22/21 |  | 28.6 ± 3.4 | 27.9 ± 3.5 |  |
| Molt, *et al* (2014)[39] | 70.0 ± 7.0 | 67.0 ± 9.0 |  | 16/14 | 16/14 |  | 28.0 ± 3.0 | 28.0 ± 3.0 |  |
| Dennis, *et al* (2015)[12]* | 62.0 ± 6.0 | 62.0 ± 6.0 |  | 16/12 | 16/12 |  | 29.0 ± 4.0 | 29.0 ± 4.0 |  |
| Ejaz, *et al* (2015)[14] | 68.3 ± 8.4 | 68.2 ± 7.8 |  | 13/16 | 15/13 |  | 25.1 ± 2.0 | 25.2 ± 2.5 |  |
| Rathod, *et al* (2014)[44] | 64.1 ± 7.3 | 63.6 ± 5.9 |  | 17/23 | 20/20 |  | 31.6 ± 7.3 | 29.1 ± 5.2 |  |
| Ayik, *et al* (2020)[8] | 65.39 ± 7.25 | 64.90 ± 6.58 |  | 14/18 | 14/19 |  | 31.38 ± 4.72 | 30.31 ± 7.10 |  |
| Chaudhry, *et al* (2020)[10] | 62.29 ± 9.63 | 65.41 ± 9.04 |  | 51/66 | 57/66 |  | 30.18 ± 0.69 | 30.81 ± 2.09 |  |
| Hedge, *et al* (2021)[20] | 63.64 ± 7.02 | 63.66 ± 7.26 |  | 37/24 | 37/24 |  | 30.43 ± 4.81 | 29.48 ± 5.15 |  |
| Pinsornak, *et al* (2021)[42] | 67.8 ± 8.1 | 69.2 ± 7.5 | 65.7 ± 7.6 | 9/41 | 8/42 | 10/40 | 24.8 ± 2 | 25.2 ± 2.5 | 24.8 ± 2 |
| YiZ, *et al* (2021)[55] | 68.44 ± 6.8 | 68.7 ± 7.3 | 68.00 ± 7.11 | 7/43 | 7/43 | 8/42 | 26.13 ± 2.63 | 25.88 ± 3.51 | 25.34 ± 3.61 |
| Zhao, *et al* (2020)[58] | 65.01 ± 9.59 | 65.55 ± 7.87 | 64.53 ± 8.54 | 37/23 | 42/18 | 40/20 | 26.35 ± 3.52 | 25.72 ± 4.78 | 25.74 ± 5.41 |

^1^tourniquet use/ long duration tourniquet use; ^2^tourniquet use/ short duration use; ^3^tourniquet use with tranexamic acid

SD= Standard Deviation; m/f= male/female; BMI= Body Mass Index, NI= No information

*The study randomized the right and left leg of one group of participants.


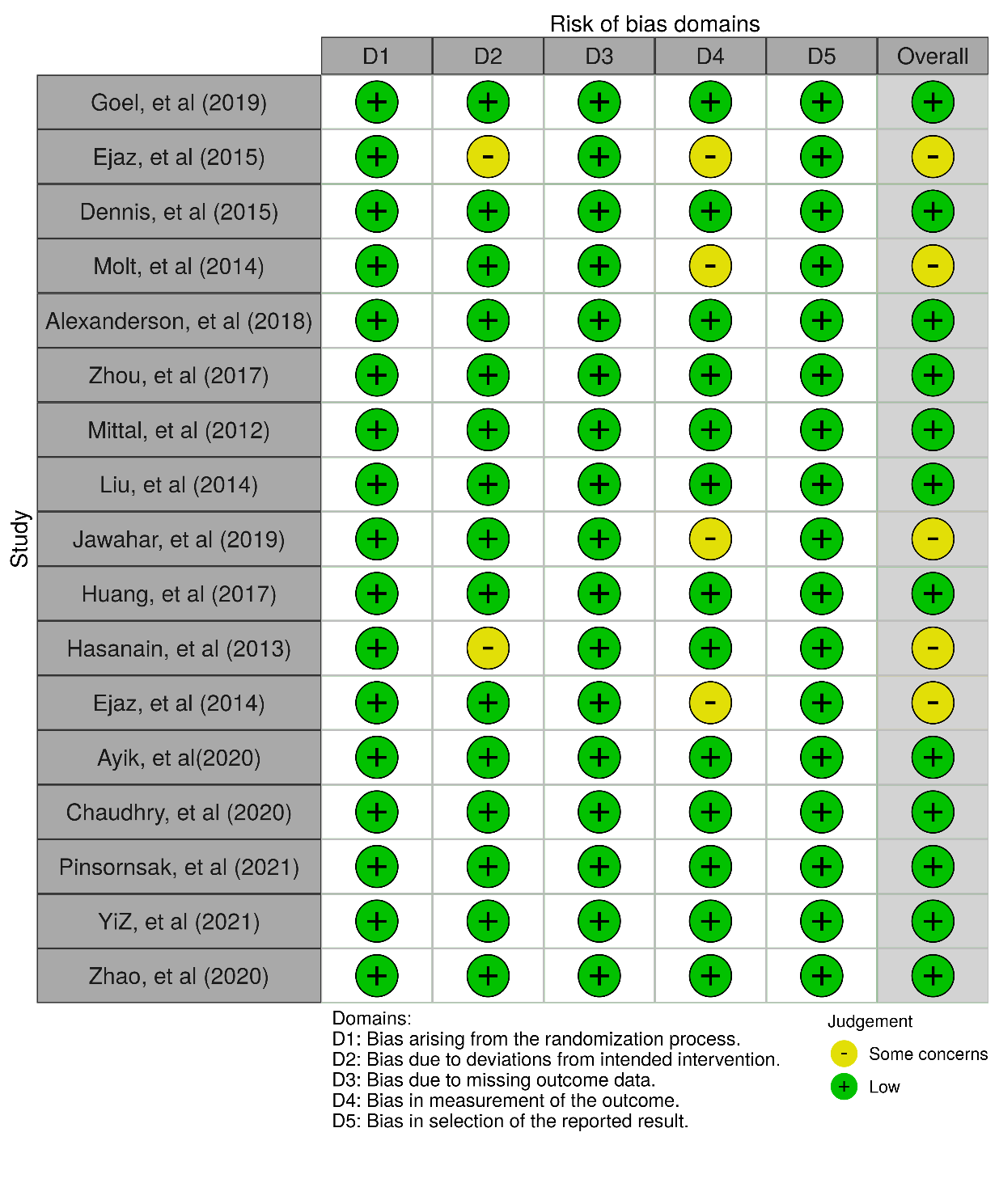
Appendix 3: Quality assessment of RCTs using Risk of Bias Assessment tool for review assessing the long-term effects of tourniquet use in TKA

Appendix 4: Quality assessment of cohort studies using Newcastle-Ottawa scale for review assessing the long-term effects of tourniquet use in TKA

| Study Details | Selection |  |  |  | Comparability* | Outcome** |  |  | Total (9*) |
| --- | --- | --- | --- | --- | --- | --- | --- | --- | --- |
|  | Representativeness of exposed cohort ( ) | Selection of non-exposed cohort ( ) | Ascertainment of exposure ( ) | Absence of outcome at start ( ) | ( ) | Assessment of outcome ( ) | Duration of follow-up ( ) | Adequacy of follow-up ( ) |  |
| Touzopoulos, et.al (2019)[52] |  |  |  |  |  |  |  |  | 9 |
| Rathod, et.al (2014)[44] |  |  |  |  |  |  |  |  | 8 |
| Ajnin, et.al (2019)[4] |  |  |  |  |  |  |  |  | 7 |
| Hedge, et.al (2021)[20] |  |  |  |  |  |  |  |  | 9 |

***** Comparability assessed as the following: one star rewarded if study controlled for the severity of disease of the subjects, another star rewarded if study adjusted or controlled for age, gender, BMI and protocol of surgery.

**Adequacy of follow-up was assessed as the following: one star rewarded if all subjects accounted or if subjects lost to follow up unlikely to introduce bias - small number lost - >80 % subjects present
